# LncRNA XIST acts as a MicroRNA-520 sponge to regulate the Cisplatin resistance in NSCLC cells by mediating BAX through CeRNA network

**DOI:** 10.7150/ijms.49730

**Published:** 2021-01-01

**Authors:** Ting-Ting Liu, Rui Li, Xiao Liu, Xi-Jia Zhou, Chen Huo, Jian-Ping Li, Yi-Qing Qu

**Affiliations:** 1 Department of Pulmonary and Critical Care Medicine, Qilu Hospital, Cheeloo College of Medicine, Shandong University, Jinan 250012, China.; 2 Department of Pulmonary and Critical Care Medicine, Qilu Hospital of Shandong University, Jinan 250012, China.

**Keywords:** ceRNA, LncRNAXIST, biomarker, BAX, apoptosis

## Abstract

**Background:** In recent years, LncRNA acts as a member of competing endogenous RNA (ceRNA), playing an important role in drug resistance of lung cancer. The aim of this study was to identify potential biomarkers about cisplatin resistant lung cancer cells using a comprehensive ceRNA network.

**Methods:** GSE6410 (GPL-201) analyzed gene expression changes about cisplatin resistance in A549 NSCLC cells. GSE43249 (GPL-14613) included noncoding RNA expression profiling derived from the cisplatin resistant A549 lung cells. GEO2R, an online analysis tool, analyzed the differentially expressed mRNAs and miRNAs (DEmRNAs and DEmiRNAs). To explore the functional enrichment implication of differentially expressed mRNAs, we used the GO (Gene ontology) and KEGG (Kyoto Encyclopedia of Genes and Genomes) pathway analysis. Through miRDB, Targetscan, Starbase and miRWalk, we found targeted miRNAs. The Kaplan-Meier curve method was used to show clinical survival analysis of targeted RNAs (*P<0.05*). The Starbase database predicted potential lncRNAs mediated targeted miRNAs. Eventually, the novel ceRNA network of lncRNAs, miRNAs, mRNA was constructed by cytoscape3.7.2.

**Results:** 118 differentially expressed mRNAs were the basis of the mediated ceRNA network. DAVID and Kaplan-Meier picked out BAX, an apoptosis regulator. Venn diagram demonstrated 8 miRNAs commonly regulating BAX. Starbase predicted lncRNA XIST mediated miRNAs. Finally, lncRNA XIST may be a useful biomarker regulating cisplatin resistance in lung cancer cells and further, we explored the BAX may effect tumor-infiltrating immune cells.

**Conclusions:** LncRNA XIST competitively bound to miRNA 520 in the regulation of cisplatin resistance by BAX, participating apoptosis in the p53 signaling pathway.

## Introduction

Lung cancer is considered to have a significant role in cancer-related deaths worldwide. It is well known that NSCLC comprises approximately 85% of all lung cancers and lung adenocarcinoma (LAC) accounts for 40% of all lung cancers all over the whole world 1.

As for NSCLC, many treatment strategies are effective and include surgical operation, chemotherapy and radiotherapy. NSCLC patients, who are treated with cisplatin, also termed cisdiamminedichloroplatinum (CDDP) or diamminedichloro platinum (DDP); may develop chemoresistance 2. Cisplatin has been adopted for about 30 years 3, primarily acting by causing DNA damage 4. Chemoresistance is a major problem for cancer therapy 5. There are many resistant mechanisms examined in cancer cells, such as the P53 signaling pathway, apoptosis 6 and the cell cycle. At present, chemotherapy has been limited due to resistance 7. However, relative biomarkers are considered absent 8. Thus, it is high time that we should establish effective network of biomarkers to predict gene changes in cisplatin resistant NSCLC cells.

LncRNAs have been considered as oncogenes mediating tumorigenesis and chemoresistance. They may have lower expression and exist in the cytoplasm or nucleus 9. They inhibit effects on miRNAs and mRNAs 10. In different kinds of cancers, a large number of lncRNAs are explored to mediate cellular processes and drug-resistance, such as NSCLC, ovarian cancer 11, gastric cancer 12, pancreatic cancer 13, and breast cancer 14. LncRNA acts as a member of competing endogenous RNAs (ceRNAs) by competitively binding targeted microRNAs (miRNAs) 15, 16. In our study, lncRNA XIST locates on chromosome 8q24.21 and combines with the microRNA-520 to regulate the cisplatin resistance by mediating BAX through ceRNA network.

BAX 17 (ENSG00000087088), an apoptosis regulator molecule 18, is located on the chromosome 19, with 13 transcripts. BAX is a member of Bcl2 family 19. DAVID database and Kaplan-Meier curve were used to analyze the GSE6410 DEmRNAs. Next, through four databases, miR-525-5P, miR-4640-3p, miR-214-3P, miR-520a-5p all regulated the BAX. Comparing with GSE43249 (GPL-14613), we decided to explore miR-520 (survival curve *P<0.01*). In this present novel, the ceRNA network of lncRNA-miRNA-mRNA about cisplatin resistance in NSCLC was created through RNA sequencing data from the GEO database.

## Materials and Methods

### Data collection and microarray analysis

We used GSE6410 (6 samples mRNAs) and GSE43249 (6 samples miRNAs) 20 about cisplatin resistant non-small cell lung cancer (NSCLC) cells from the GEO database 20 (https://www.ncbi.nlm.nih.gov/gds/?term=). The inclusion criteria were as follows. Firstly, our goal was to find mRNA and noncoding RNA expression changes about cisplatin resistance in A549 NSCLC cells from GEO database. Through some key words, such as mRNA, noncoding RNA, cisplatin, lung cancer, we screened targeted datasets. Next, for better comparison, we used data including A549 cisplatin-sensitive and cisplatin-resistant non-small cell lung cancer cell lines. The exclusion criteria were incomplete data, which didn't have control group. First, DEmRNAs were analyzed by online software GEO2R. There were 8793 DEmRNAs in GSE6410, and |Log 2 FC|>1 and the *P* <0.05 21 were two screening criteria. These 118 DEmRNAs were the basis of ceRNA network. GraphPad-Prism 8.4.0 (https://www. graphpad.com) 22 was used to show volcano maps and heat maps. To normalize the samples, we showed Box diagram vividly. The Independent two-samples T-test proved that samples were representative by SPSS Statistics 17.0.

### Functional enrichment analysis of DEmRNAs

The DAVID database 6.8 (https://david.ncifcrf.gov/) 23 and Metascape (http://metascape.org) 24 were used to find the function and pathways of 118 DEmRNAs. As to GO-BP (biological process), sorted by *P* value <0.05, 5 counts (CDKN1A, BTG2, BAX, MDM2, and GADD45A) were most significant in participating DNA damage response, p53 signaling pathway. To GO-CC (Cellular component), calcium channel complex was numerous. Steroid binding accounted most in the GO-MF (molecular function). All genes in the genome have been used as the enrichment background. Through the Kaplan Meier-plotter software (https://kmplot.com) 24, we finally decided to study the targeted mRNA BAX, an apoptosis regulator molecule, which participated in the P53 signaling pathway 25.

### Explore potential miRNAs

According to the targeted molecule BAX, we found miRNAs, which commonly regulated BAX from miRDB 26 (http://www.mirdb.org), Targetscan 26 (http://www.targetscan.org), Starbase 27 (https://starbase.sysu.edu.cn/), miRWalk (http://mirwalk.umm.uniheidelberg.de/) 28. Then, we showed 8 miRNAs regulating BAX in only four databases by Venn software, such as hsa-miR-3681-3p, hsa-miR-766-5p, hsa-miR-525-5p, hsa-miR-4640-3p, hsa-miR-128-3p, hsa-miR-216a-3p, hsa-miR-520a-5p, hsa-miR-214-3p. But these miRNAs were not all related to the survival of patients. Later, 4miRNAs (hsa-miR-4640, hsa-miR-520a-5p, hsa-miR-214-3p, hsa-miR-525-5p) were obviously related to overall survival 29 (*P*<0.05) by the Kaplan Meier plotter software (https://kmplot.com) 24.

### The discovery of LncRNA XIST in cisplatin resistant NSCLC cells

Identification of potential lncRNA about 4 miRNAs (hsa-miR-4640, hsa-miR-520a-5p, hsa-miR-214-3p, and hsa-miR-525-5p) was the critical step. The Starbase database (https://starbase.sysu.edu.cn/) 27 was used to separately predict potential lncRNAs for 4 miRNAs. Two lncRNAs XIST, MIR29B2CHG were found to regulate common 4 miRNAs by the Dram-Venn-Diagram (http://bioinformatics.psb.ugent.be/webtools/Venn/) 30 database. According to the present articles, lncRNA XIST has some researches in the cisplatin resistant NSCLC cells. The expression of lncRNA-XIST was significantly higher in NSCLC tumor tissues and cisplatin resistant A549 LAC cells 31. At the moment, research has found that lncRNA XIST was upregulated and induced chemoresistance by combing with miR-29c participating in the DNA repair pathway 32. However, the ceRNA network mediated by lncRNA XIST remained poorly understood. Thus, our study explored lncRNAXIST to predict the prognosis of NSCLC patients. This molecule may be a potential target for the cisplatin resistant lung cancer cells.

### Construction of lncRNAs-miRNAs-mRNAs network in cisplatin resistant NSCLC cells

**Figure 1:**
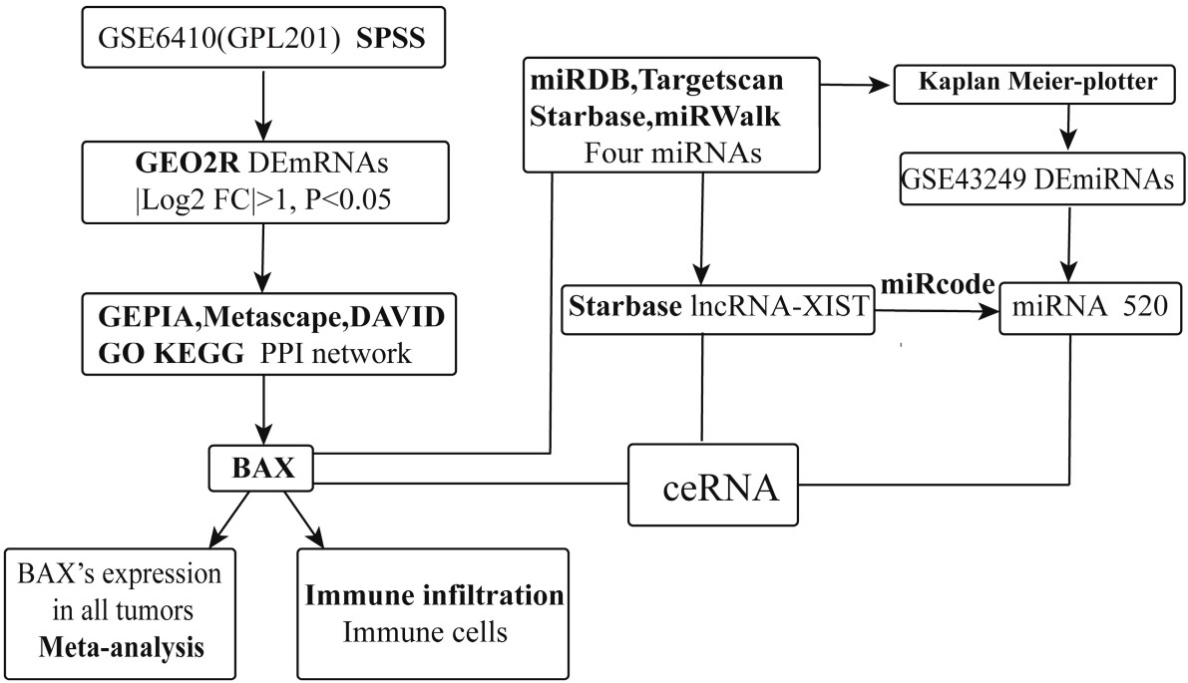
The detailed process of constructing a ceRNA network about lncRNAs- miRNAs - mRNA.

PPI networks can provide information on the molecular mechanism. The same to us, lncRNA-miRNAs-BAX network were established by cytoscape3.7.2 software obviously (http://www.cytoscape.org/) 24. Every type of RNAs all represented different nodes and the relationships between these genes were considered to be edges. In this circumstance, BAX, 4miRNAs, lncRNA XIST were all visualized in this ceRNA family. LncRNAXIST regulated the miRNAs by BAX, participating in P53 signaling pathway in cisplatin resistant NSCLC (**Figure 1**).

### Database validation of the most likely miRNA

Contrarily, through the database miRcode 33 (http://www.mircode.org), we predicted potential miRNA by lncRNA XIST. An miRNA family including miRNA 520 and miRNA 214 were also expressed in the ceRNA network. To recognize the differentially expressed miRNAs, GEO2R, an online software was used to conclude the primary data in GSE43249 (*P* value <0.05 and |Log2FC| >1). Besides, according to the differentially expressed miRNAs in the GSE43249 (GPL14613), we found out miRNA520 in NSCLC. This further proved the importance of miRNA520 in the cisplatin resistant NSCLC cells. Thus lncRNA XIST, miRNA520, BAX were considered to be the key molecules, mediating the apoptosis in the P53 signaling pathway.

## Results

### Differentially expressed mRNAs in NSCLC

**Figure 2:**
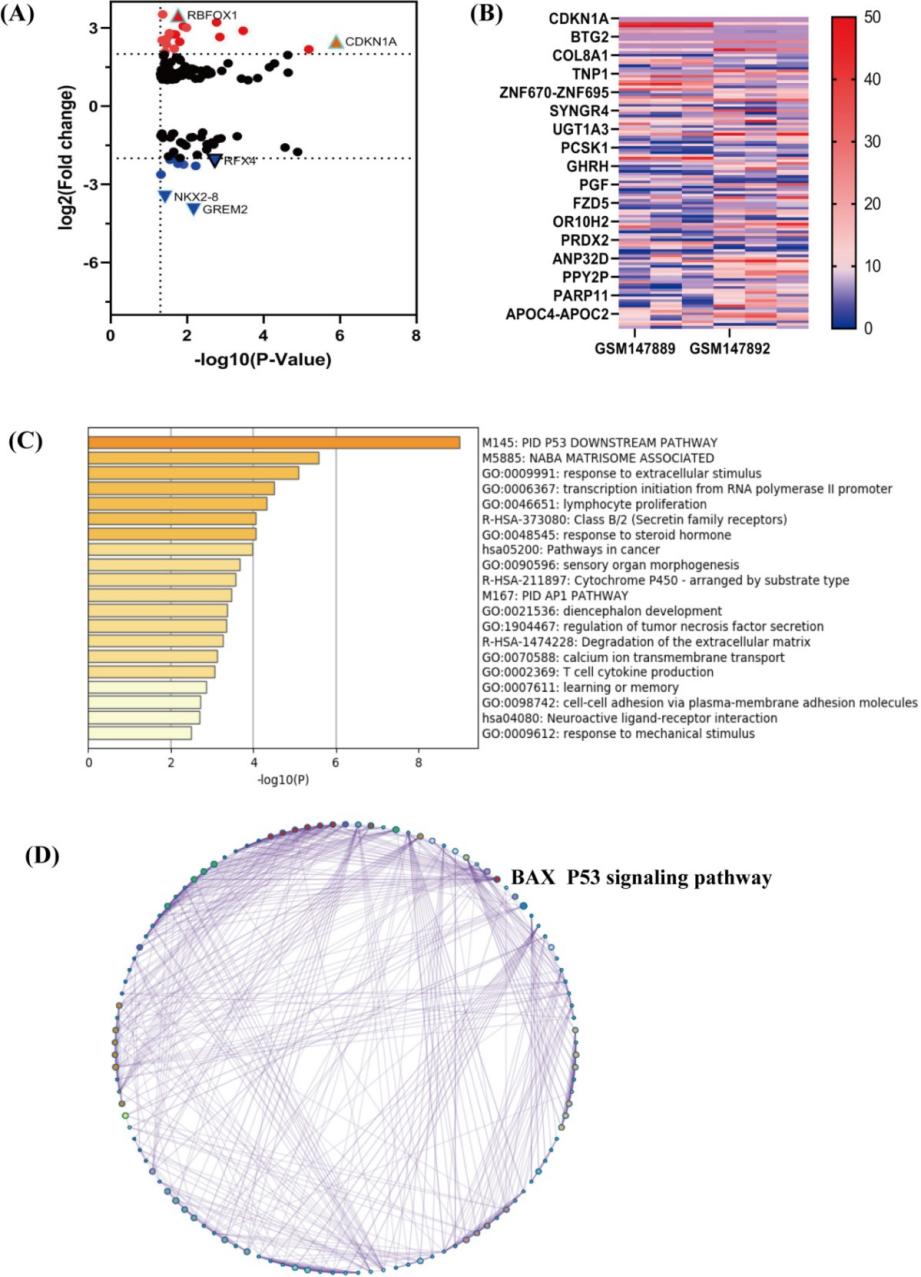
** The map and enrichment analysis of DERNAs. (A)** Volcano map of differentially expressed mRNAs in cisplatin sensitive-resistant NSCLC, red represents up-regulated mRNAs, 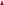 is the most obvious in mRNAs, 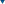 is considered to be down-regulated significantly; **(B)** Heat map of DEmRNAs in NSCLC. The red represents more expression in samples. **(C)** GO enrichment results of 114 (118) DEmRNAs, P53 pathway is remarkable. The size of the rectangle represents the number of enrichment analysis participants. **(D)** Network of enriched terms: colored by cluster ID, the red is the molecule BAX. All nodes are typically close to each other.

Through comparison and screening, we chose GSE6410 (GPL201) including mRNAs about cisplatin resistant cancer cells. GEO2R, an online software analyzed 8793 DEmRNAs from the samples (absolute Log Fold change>1 and *p*-value <0.05). There were 118 DEmRNAs, including 80 up-regulated genes, 38 down-regulated genes. In order to show DEmRNAs, we used the volcano maps and heat maps (**Figure 2A, 2B**). We chose top 25 down regulated and up regulated mRNAs (**Table 1**).

### Enrichment Analysis and pathway of DEmRNAs

We used the Metascape (http://metascape.org), an online software, to figure out the functions and pathways of 118 DEmRNAs. The most significant term was shown to represent the cluster (**Figure 2C**). The p53 signaling pathway had numerous counts, including the targeted biomarker BAX. To show pathways of DEmRNAs, the enriched terms were constructed as a network. The different colors were classified by cluster ID, and the common cluster ID was closely related to each other (**Figure 2D**).

**Figure 3:**
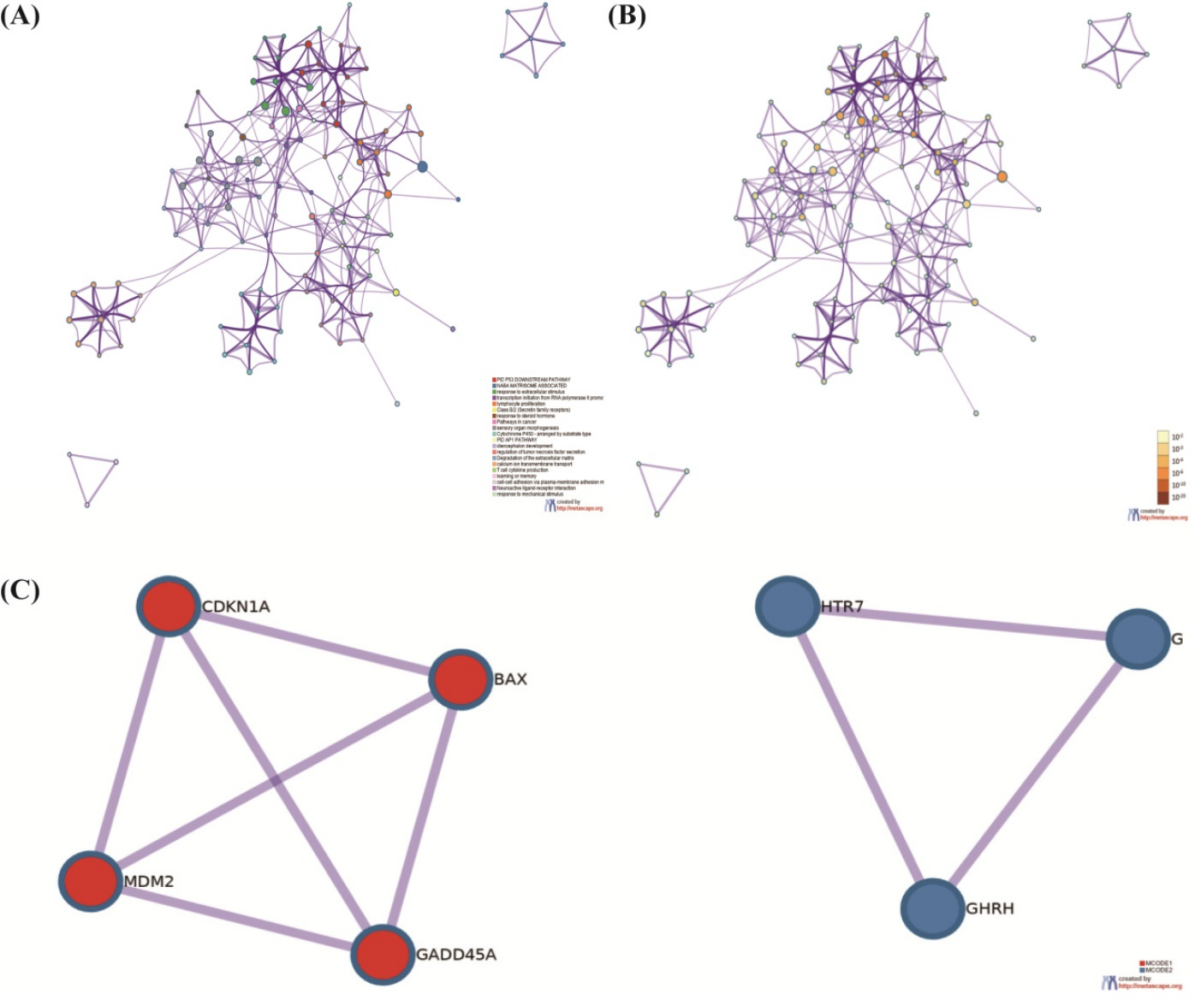
** Network of enriched terms by metascape software. (A)** Colored by cluster ID, nodes are different terms. **(B)** Colored by p-value. The significant p-value represents that terms contain more genes. **(C)** PPI network and MCODE components identified in the gene lists. The red represents mRNAs have good correlation and mediate in the P53 signaling pathway, including the targeted molecule BAX.

At the same time, the top 5 terms of enrichments were listed as follows (**Table 2**). Through the GO and KEGG analysis, the terms of P53 signaling pathway were typical. DEmRNAs may take part in this pathway to enhance cisplatin resistance in NSCLC cells. We selected the terms having the best *p*-values from each of the 20 clusters to show the relationships between the terms. Through the Cytoscape, we constructed the network where each node was an enriched term. Firstly, the network was colored by cluster ID (**Figure 3A**) and then by *p*-value (**Figure 3B**). We can identify that DEmRNAs were interrelate with each other. PPI networks can provide information on the molecular mechanism underlying cellular activity. In our study, a PPI network of differentially expressed mRNAs in NSCLC provided 4 mRNAs (CDKN1A, BAX, MDM2, GADD45A), which were associated with each other tightly (**Figure 3C**). They were all key biomarkers in the P53 signaling pathway.

### Identification of targeted molecule BAX

**Figure 4:**
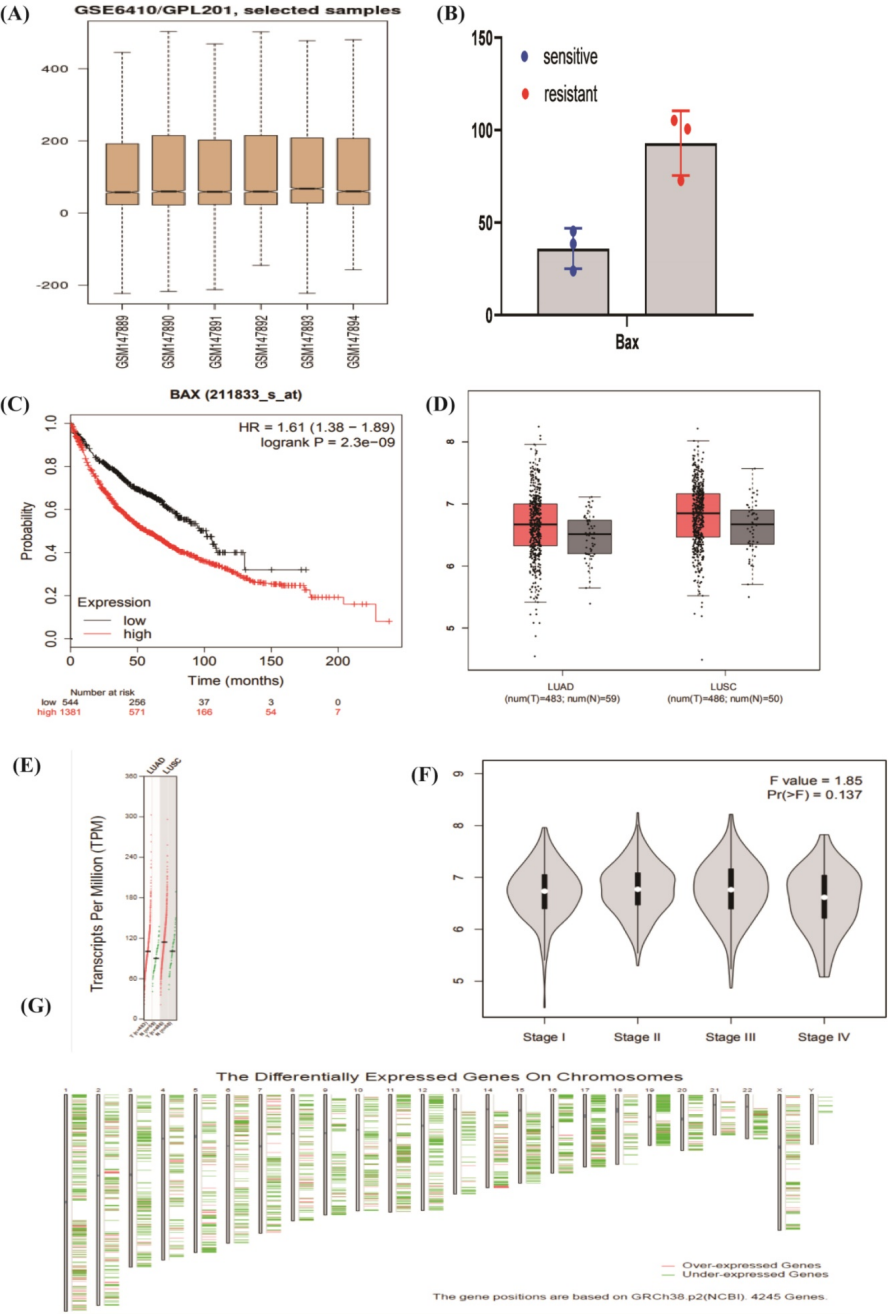
** The expression of targeted molecule BAX. (A)** The box diagram demonstrates the samples of cisplatin sensitive-resistant samples are standardized. **(B)** BAX is up-regulated obviously in cisplatin resistant NSCLC cells. Detailed research of BAX in some online databases. **(C)** BAX is associated with overall survival (*P*<0.05) in NSCLC. **(D,E)** The expression of BAX in LAC and LUSC is higher than normal cells. **(F)** BAX has little impact on patient's staging. **(G)** On the chromosome 14, some genes, associated with BAX are considered to be over-expressed typically.

Through the function enrichment analysis and pathway, we found that the counts in the P53 signaling pathway were most typical. Four mRNAs, CDKN1A, BAX, MDM2, and GADD45A; attracted our interest deeply. BAX may regulate the cisplatin resistance in NSCLC. We utilized a *t*-test to illustrate that the samples were representative (*P*-value=0.013) by SPSS17.0 34. We used the box diagram to prove that GSE6410 was normalized (**Figure 4A**). In the NSCLC, the expression of BAX was highly showed in resistant lung cancer cells (**Figure 4B**). The Kaplan Meier-plotter software (https://kmplot.com) was used to find the survival analysis of BAX (*P*<0.01) (**Figure 4C**). By the GEPIA database (http://gepia.cancer-pku.cn/index.html), BAX's expression Profiling was higher in the LAC and LUSC cancer cells (**Figure 4D, 4E**). As for cancers, different stages represent people's prognosis. Through our study, we proved that BAX had little impact on patients' staging (**Figure 4F**). Some genes which were similarly with BAX, maybe up-regulated or down-regulated on chromosome (**Figure 4G**). From the graph, some genes were considered to be over-expressed typically on the chromosome 14.

### Searching of potential lncRNAXIST-miRNAs-BAX

**Figure 5:**
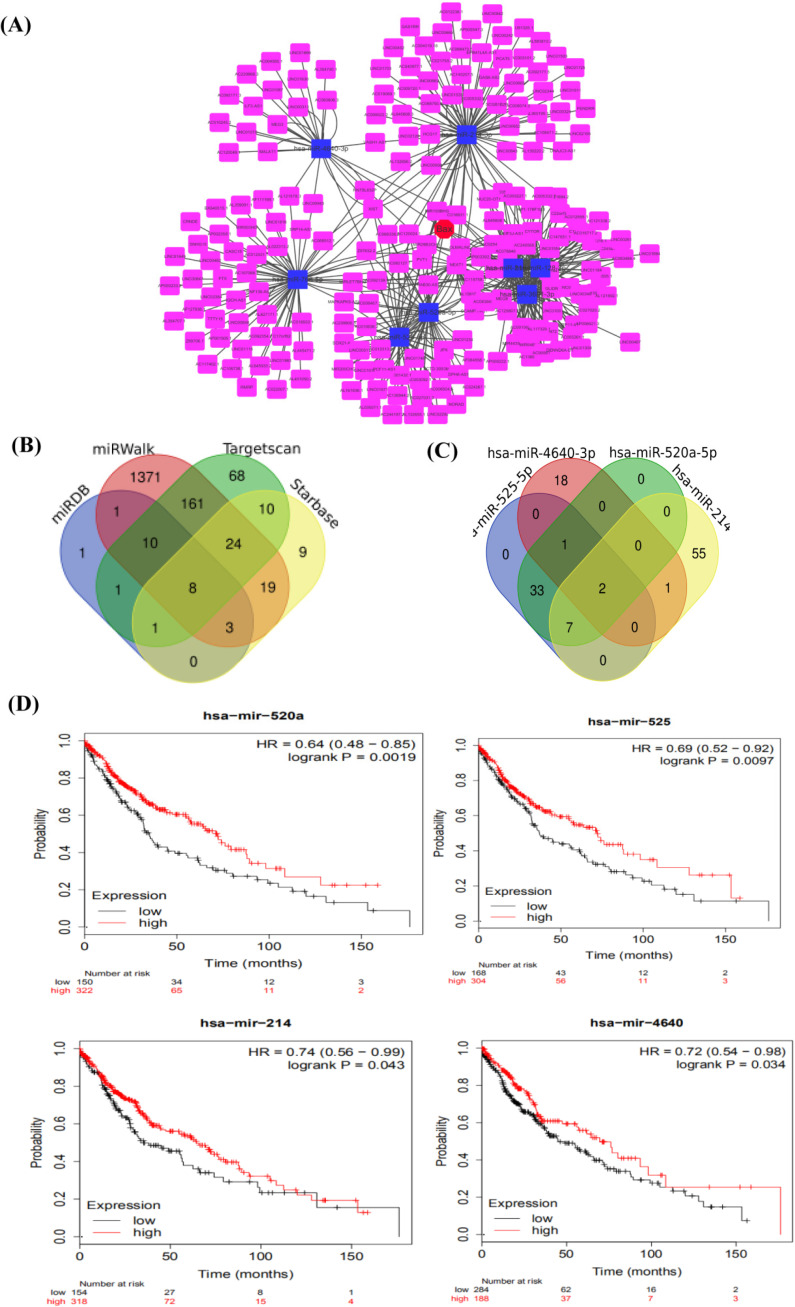
** The construction of ceRNA in NSCLC. (A)**The ceRNA network of lncRNA-8miRNAs-BAX, red is the BAX, blue is miRNAs, pink is lncRNAs. **(B)** The combination of miRNAs and lncRNAs. Four kinds of databases are mixed to explore miRNAs. **(C)** Looking for the common lncRNA of 4miRNAs. Two common lncRNAs are XIST and MIR29B2CHG. **(D)** The OS (overall survival) analysis of 4 miRNAs in NSCLC patients using Kaplan Meier curve (*P*<0.05).

As is shown above, the BAX may be a useful molecule to cisplatin resistance. We chose the BAX as the important molecule to find miRNAs through different databases, including miRDB (http://www.mirdb.org); Targetscan (http://www.targ-etscan.org); Starbase (https://starbase.sysu.edu.cn/); and, miRWalk (http://mirwalk.umm.uni-heidelberg.de/). 8 miRNAs, miR-3681-3p, miR-766-5p, miR-525-5p, miR-4640-3p, miR-128-3p, miR-216a-3p, miR-520a-5p, and miR-214-3p were expressed commonly by the Dram-Venn-Diagram (http://bioinformatics.psb.ugent.be/webtools/Venn/) (**Figure 5B**). The ceRNA network of lncRNA XIST-8 miRNAs-BAX were showed in the map (**Figure 5A**) and we used the cytoHubba to choose the Hub gene. In the NSCLC, we predicted the survival of 8 miRNAs by Kaplan-Meier-Plotter, but only 4 miRNAs significantly affected quality of people's life and longevity (**Figure 5D**). Using the starbase, we found lncRNAs of these 4 miRNAs and selected out together expressed lncRNA (**Figure 5C**). LncRNA XIST and MIR29B2CHG may be the targeted ceRNAs. Combining with some articles reported in recent years, lncRNA XIST has some researches in cisplatin sensitive-resistant NSCLC cells. We considered that lncRNA XIST may act as a miRNA sponge to lead cisplatin resistance. At the same time, we combined the GSE43249's differentially expressed miRNAs, 2 miRNAs were included, such as miR-520, miR-525. miRcode database was used to verify miRNAs from lncRNA XIST. MiR-520, miR-525 were targeted miRNAs of lncRNA XIST. In the present study, lncRNA XIST acted as a member of ceRNA, competitively bound miRNA520 to regulate the BAX, apoptosis associated X, in the P53 signaling pathway.

### The correlation between hub genes and immune cell infiltration

**Figure 6:**
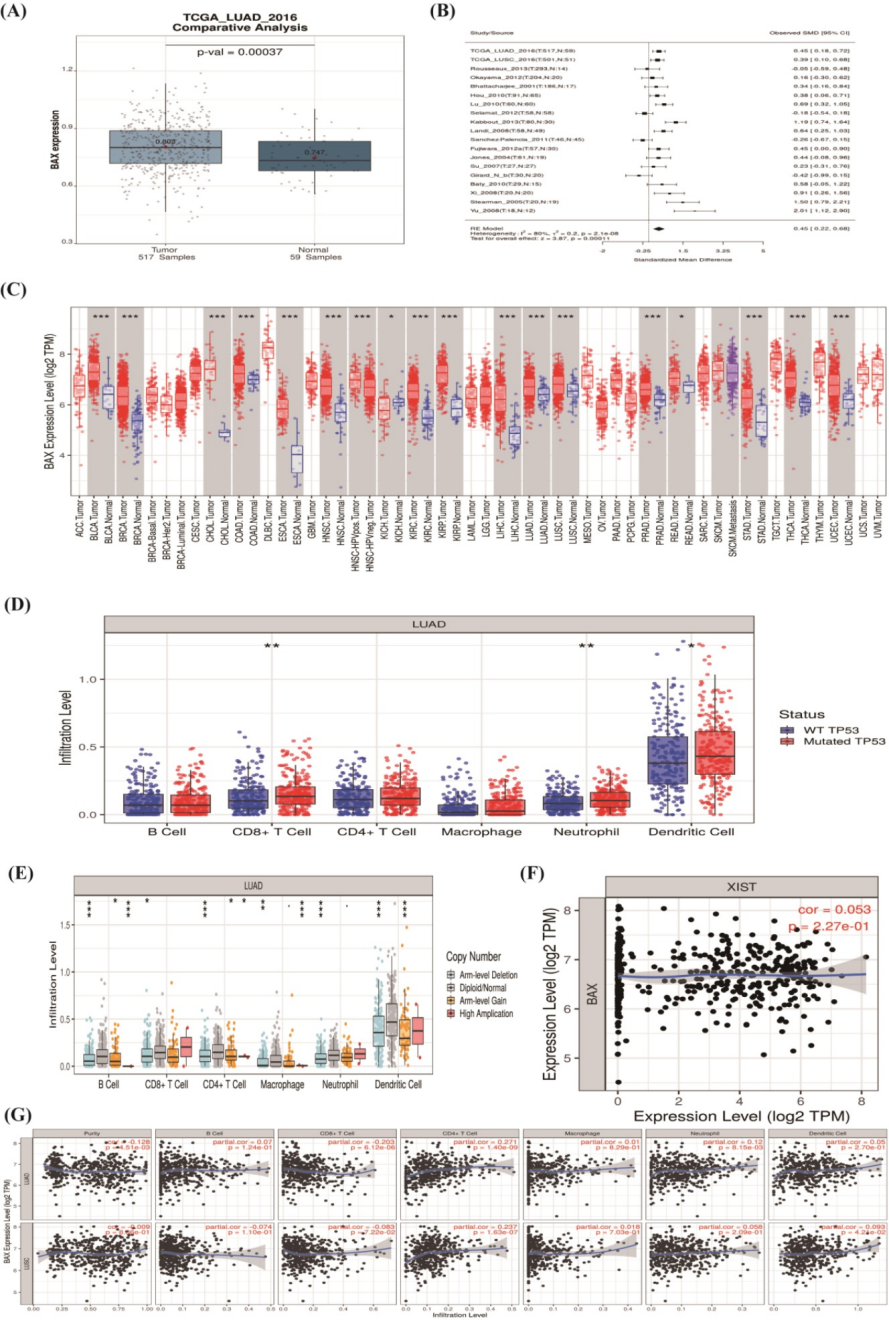
** The correlation between BAX and immune cell infiltration. (A)**The expression of BAX in 517 tumor samples than 59 normal samples (*P*=0.0037). **(B)** BAX's expression was significant through meta-analysis. **(C)** BAX expression level was commonly higher in all tumors. **(D,E)** The mutation of TP53 and BAX all influence immune cells (*P*<0.05). **(F)** LncRNA XIST and targeted molecule BAX's expressions were positive correlation (cor=0.053,P=2.27e-01). **(G)** The positive correlations between BAX and immune cells in LAC and LUSC.

In order to verify the accuracy of the data, we used the The Cancer Genome Atlas (TCGA) database through Lung cancer explore (LCE) database (http://lce.biohpc.swmed.edu/lungcancer/index.php#page-top). The expression of BAX was higher in 517 tumor samples than 59 normal samples (*P*=0.0037) (**Figure 6 A**). In different types of lung cancer, we found BAX's expression was significant through meta-analysis (**Figure 6B**). At the moment, BAX expression level was commonly higher in all tumors through the TIMER database (https://cistrome.shinyapps.io/timer/) (**Figure 6C**). Details were given in **Table 3.** In recent years, the tumor immune infiltration has taken important roles in cancer. The mutation of TP53 and BAX all influence immune cells; such as the B cell, CD8+ T cell, CD4+ T cell, macrophage, neutrophil, and dendritic cell (*P*<0.05) (**Figure 6D, 6E**). Our results revealed positive correlations between BAX and immune cells in LAC and LUSC (**Figure 6G**). LncRNA XIST and targeted molecule BAX's expressions were positive correlation (cor=0.053, *P*=2.27e-01) (**Figure 6F**).

## Discussions

Lung cancer represents a common cancer all over the world and lung adenocarcinoma (LAC) is the most numerous type, with high diversity 35. At present, there are many treatments for different types of cancer, such as surgical operation, chemotherapy, and so on. With the development of molecule-targeted drugs, the molecule-targeted treatment of tumors has been widely agreed. However, due to various reasons, a large number of patients are not sensitive to drugs. Because of complex molecular mechanisms, multiple drug resistance may lead chemotherapeutic failure for lung cancer patients 36.

DDP is a significant chemotherapeutic drug in lung cancer. Thus, the potential mechanism of chemical resistance remains necessary to determine 37. Therefore the further understanding of resistant theory is helpful for choosing chemotherapy drugs 38.

In recent years, lncRNAs have been explored through functional analysis 39. More importantly, lncRNA acts as a member of ceRNA, participating in many functions in the field of cancer, such as protein modification, cell proliferation. Cell apoptosis, a classic signaling pathway, participates in cancer Proliferation and drug resistance.

Our study explored the role of potential lncRNA XIST, acting as a ceRNA of miR520 by regulating BAX involving in the P53 signaling pathway. Through the enrichment analysis of 118 DEmRNAs, we found that the targeted molecule BAX had higher expression in DDP. Four online databases were used to identify potential miRNAs mediated BAX. LncRNA XIST regulated four valuable miRNAs by starbase database.

**Figure 7:**
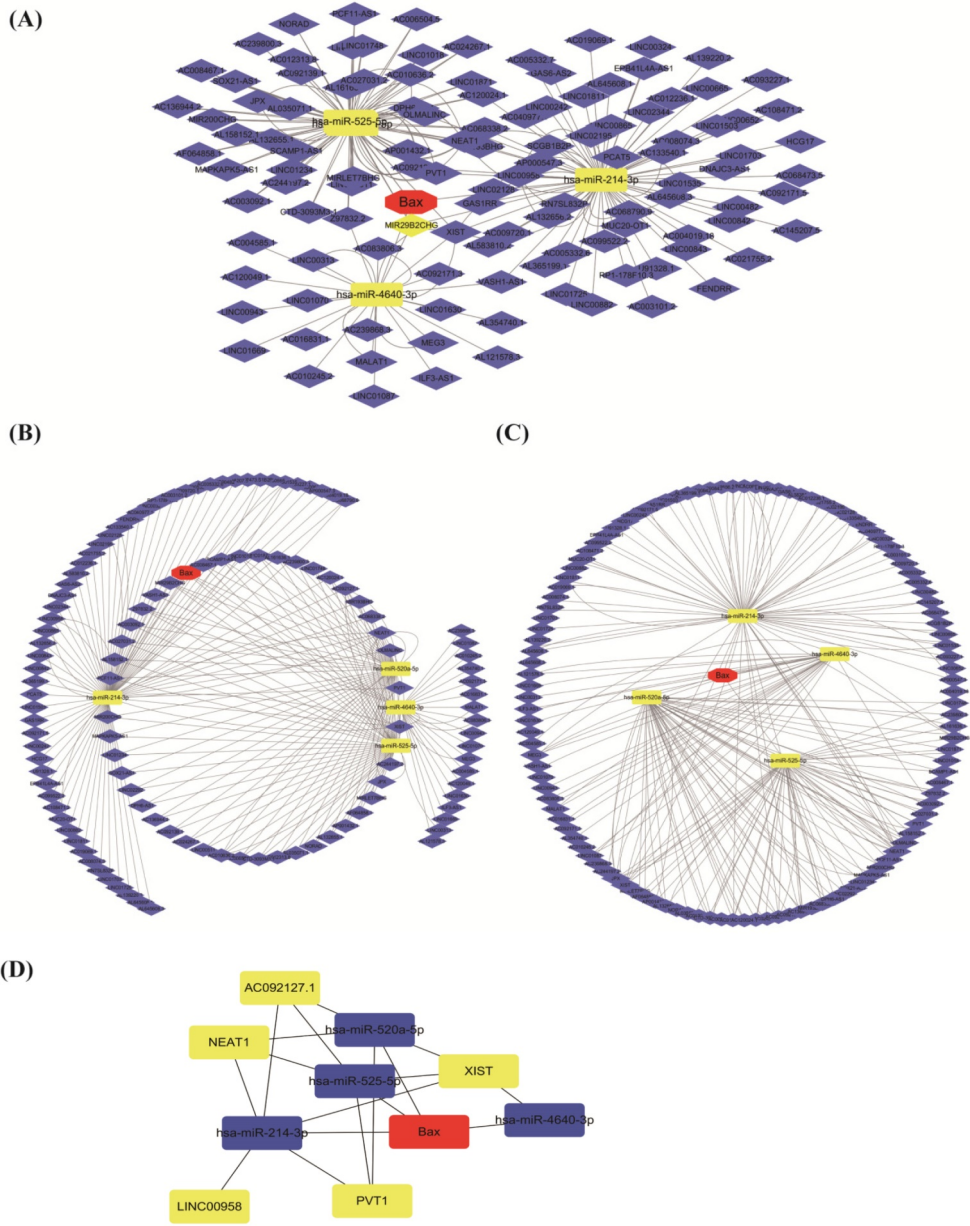
** The ceRNA network of lncRNA XIST-miRNAs-BAX by cytoscape. (A, B, C)** The red represents the BAX, the yellow is miRNAs and the blue is lncRNAs. **(D)** Through the cytoHubba, we select top 10 RNAs with higher degree. Different colors show different RNAs.

The present findings clearly demonstrate that the expression of long non-coding RNA (lncRNA XIST) is up-regulated in lung cancer cells, and it maybe involve in the cell proliferation and TGF-β1-mediated apoptosis 38. And lncRNAXIST may have some functions by downregulating miRNA-144. Compared with previous studies, the present study eventually constructed ceRNA 40 network of lncRNA XIST-miRNAs-BAX. We selected top 10 hub RNAs with higher degree through the cytoHubba (**Figure 7A-D**).

The implications of the research were providing some new treatment ideas. We found that molecules BAX, miRNA520, lncRNA XIST have been regulated closely each other. In addition, cisplatin can induce cell death by engaging endogenous apoptotic signaling that activates mitochondrial apoptosis 8. We found that induction of p53 promoted apoptosis, autophagy, and cell sensitivity to cisplatin treatment 41. Our study provided new insight into the mechanisms underlying chemoresistance of lung cancer 42. Our study found an important role for LncRNA XIST in cisplatin resistance, acting as a ceRNA regulatory pathway in NSCLC that may be effective for preventing chemoresistance. In the future, researchers can use these promising targeted biomarkers to avoid cisplatin resistance.

For different tumors, the relations of long noncoding RNA (lncRNA) and microRNA influence cell process, cell growth, and drug resistance 43, 44. We all know that miRNAs regulate their genes to perform different functions 45. For lung cancer, miRNAs have numerous functions, such as tumor cell proliferation and progression, inflammation 46. A previous study has showed that miR‑1284 influences apoptosis of lung tumorigenesis 47. BAX is associated with apoptosis in cancer cells through some reports, and it participates in the classic P53 signal pathway. We predicted different molecules through individual databases. This study firstly explored 8 potential miRNAs. Later after analysis, only 4 miRNAs were associated with patients' survival (*P*<0.05). Combining with the GSE43249 and miRcode database, miR520 was a potential molecule by regulating BAX in cisplatin sensitive-resistant NSCLC cells. Through the TIMER database 48, 49, we explored the BAX may effect tumor-infiltrating immune cells (B cell, CD8+ T cell, CD4+ T cell, macrophage, neutrophil, and dendritic cell).

Immunotherapy has achieved unprecedented success in the treatment of cancer 50. In our research, targeted biomarkers BAX and XIST all influenced immune cells, such as the B cell, CD8+ T cell, CD4+ T cell, macrophage, neutrophil, and dendritic cell (*P*<0.05). The interaction between these RNAs may have an important regulatory role in the immune infiltration in NSCLC, thereby affecting the patient's prognosis and immunotherapy efficacy.

Through our study, potential molecules maybe provided for DDP. In some way, this can reduce cisplatin resistance to chemotherapy and be benefit of clinical NSCLC patients. Of course, there are certain limitations in present study. These associated molecules were predicted only in theoretical aspects. At present, the corresponding experimental verification is lacking. At the same time, the research of molecular mechanism was not deep enough. More importantly, the present novel indicated that lncRNAXIST-miRNA 520-BAX influence cisplatin resistance in NSCLC cells.

## Conclusion

In conclusion, BAX and LncRNA XIST were up-regulated in NSCLC patients. LncRNA XIST may act as a miRNA520 sponge by regulating BAX, associated apoptosis X, through activating in the P53 signaling pathway to affect cisplatin resistance. To summary, based on our data, this may enrich the effective therapeutic methods for cisplatin resistant NSCLC patients.

## Figures and Tables

**Table 1:** The differentially expressed mRNAs in the NSCLC, top 25 down and up regulated mRNAs

Gene.symbol	logFC	*P*-value
IGLL1	-1.5812651	0.00002736
KCNMA1	-1.1513622	0.00048796
COL15A1	-1.2280541	0.00129318
SEPT5-GP1BB	-1.2577165	0.00158965
PPBP	-1.4687682	0.00202229
RFX4	-2.0975544	0.00189187
SLIT3	-1.387397	0.0022444
NR1H4	-1.4536816	0.00299161
F2RL3	-1.6589632	0.00308639
GMDS	-1.0053824	0.00390183
ATXN1	-1.1879763	0.00431833
HTR7	-1.8627858	0.00541648
IL12A	-2.289003	0.00596437
REG1CP	-6.3655607	0.00114347
ZBTB48	-1.1855046	0.00714396
GREM2	-3.9641684	0.00679631
UGT1A3	-1.1995722	0.00804717
SLC25A21	-1.5112204	0.01079803
HTN1	-2.2367784	0.01239841
TNS1	-1.431632	0.01284794
IFIH1	-1.380793	0.01468609
CELA2B	-2.0812906	0.01586124
IL5	-2.1994291	0.0172905
MYOT	-1.9866327	0.01536891
CDKN1A	2.4718188	0.00000126
FAS	2.1834746	0.00000654
SESN1	1.289292	0.00002265
FDXR	1.965755	0.00002315
PPM1D	1.6388142	0.00005177
BTG2	1.4970197	0.00006986
DDB2	1.0777424	0.00014334
CYP39A1	1.0008984	0.00025609
XPC	1.056704	0.00037856
MDM2	1.6441189	0.00084947
GADD45A	1.3567254	0.00123572
COL8A1	2.6565612	0.00139461
CYP4F11	2.8957083	0.00033948
TNP1	1.2582715	0.00236534
ATF3	1.3156861	0.0024596
RGS9	1.2250779	0.00281468
BAX	1.4006089	0.00303574
ZNF670-ZNF695	1.1936179	0.00394263
ADGRE2	1.3658147	0.00435895
NRXN3	1.0653136	0.00465841
RYR1	1.4004822	0.00621905
SYNGR4	1.0586675	0.00599735
MLNR	3.2174996	0.00169798
FOXE3	1.4357355	0.00719558
SPIN2A	1.4427327	0.00800466

**Table 2:** Representative enriched terms."Log10(P)" represents the value of different terms

GO	Category	Description	Log10(P)
M145	Canonical Pathways	PID P53 DOWNSTREAM PATHWAY	-8.99
M5885	Canonical Pathways	NABAMATRISOME ASSOCIATED	-5.58
GO:0009991	Biological Processes	Response to extracellular stimulus	-5.09
GO:0006367	Biological Processes	RNA polymerase II promoter	-4.5
GO:0046651	Biological Processes	Lymphocyte proliferation	-4.32

**Table 3:** The differential expression of BAX between tumor and adjacent normal tissues across all TCGA tumors

Tumor	Nor	*p*
BLCA.Tumor	BLCA.Normal	2.95E-08
BRCA.Tumor	BRCA.Normal	2.70E-47
CHOL.Tumor	CHOL.Normal	4.29E-08
COAD.Tumor	COAD.Normal	0.000245234
ESCA.Tumor	ESCA.Normal	1.48E-07
HNSC-HPVpos.Tumor	HNSC-HPVneg.Tumor	5.08E-08
HNSC.Tumor	HNSC.Normal	1.35E-19
KICH.Tumor	KICH.Normal	0.01868825
KIRC.Tumor	KIRC.Normal	1.07E-30
KIRP.Tumor	KIRP.Normal	2.24E-17
LIHC.Tumor	LIHC.Normal	1.28E-22
LUAD.Tumor	LUAD.Normal	1.03E-05
LUSC.Tumor	LUSC.Normal	0.000144932
PRAD.Tumor	PRAD.Normal	6.39E-14
READ.Tumor	READ.Normal	0.010291591
SKCM.Tumor	SKCM.Metastasis	0.341891447
STAD.Tumor	STAD.Normal	4.40E-09
THCA.Tumor	THCA.Normal	1.00E-28
UCEC.Tumor	UCEC.Normal	7.93E-09

## Abbreviations

LncRNA: Long non-coding RNA
GO: Gene ontology
KEGG: Kyoto Encyclopedia of Genes and Genomes
DAVID: The annotation, visualization and integrated discovery database
NSCLC: Non-small cell lung cancer
LAC: Lung adenocarcinoma
|Log2FC|: Absolute Log Fold change
TCGA: The Cancer Genome Atlas
LUSC: Lung squamous cell carcinoma
